# Robotic waterjet wound debridement – Workflow adaption for clinical application and systematic evaluation of a novel technology

**DOI:** 10.1371/journal.pone.0204315

**Published:** 2018-09-27

**Authors:** Dominik S. Schoeb, Julian Klodmann, Daniel Schlager, Philippe F. Müller, Arkadiusz Miernik, Thomas Bahls

**Affiliations:** 1 Department of Urology, Faculty of Medicine and Medical Center—University of Freiburg, University of Freiburg, Freiburg, Germany; 2 Institute of Robotics and Mechatronics, German Aerospace Center (DLR), Weßling, Germany; NYU Langone Medical Center, UNITED STATES

## Abstract

**Objective:**

We evaluated the clinical potential of a novel robotic system for autonomous performance of waterjet wound debridement.

**Summary background data:**

Within the last decade, waterjet wound debridement has proven to be a valid alternative to the conventional approach using sharp spoons and scalpel.

**Methods:**

The DLR MIRO robot using the DLR MICA instrument for robotic surgery was adapted for actuation of an ERBEJET 2 flexible endoscopic waterjet probe. Waterjet debridement of various wound shapes and sizes using a porcine skin model was compared between this novel robotic system and a control group of human medical professionals with regard to wound area cleaned by the waterjet, off-target area, and procedural time.

**Results:**

After the wound area was registered in the robotic system, it automatically generated a cleaning path and performed debridement based on generated surface model. While the robotic system demonstrated a significant advantage for the covered wound area (p = 0.031), the average off-target area was not significantly different from human controls. Human participants had high variability in cleaning quality across users and trials, while the robotic system provided stable results. Overall procedural time was significantly lower in trials performed by humans.

**Conclusions:**

Robotic waterjet wound debridement is a promising new technological approach compared to the current clinical standard of interventional wound therapy, providing higher efficiency and quality of wound cleaning compared to human performance. Additional trials on more complicated wound shapes and *in vivo* tissue are necessary to more thoroughly evaluate the clinical potential of this technology.

## Introduction

The treatment of chronic, infected, or contaminated wounds is among the oldest surgical challenges and is intrinsic to all surgical disciplines. While a variety of new, advanced methods of wound sealing or conditioning are currently available, surgical debridement still plays an essential role in clinical practice [1]. By removing necrotic tissue and foreign bodies, surgical wound bed preparation provides an optimal environment for wound healing after closure with a skin graft or secondary suture [2]. In the last decade, waterjet technology has proven effective to achieve a surgically clean wound [3]. This technology provides a high-powered parallel waterjet, which is able to remove tissue and foreign objects similar to a surgical scalpel, while providing constant water flow to remove the accumulating debris through suction. In addition to wound cleaning, the ability of the waterjet to remove soft tissue has been advantageous for applications in hepatic, renal, and brain surgery and retroperitoneal lymph node dissection [4–7]. However, waterjet surgery has some inherent limitations. For instance, the accumulation of water may obstruct the view of the wound ground, hindering surgeons from evaluating their progress. Second, the high-pressure waterjet disperses pathogens and debris into the air, potentially creating a risk of infection for the operating surgeon. To extend the capability of waterjet surgery and overcome these disadvantages, a robotic system, consisting of the DLR MIRO versatile lightweight robot and DLR MICA versatile instrument for robotic surgery [8], has been adapted to actuate a waterjet scalpel [9]. In this study, we evaluated the capability of this robotic system for autonomous debridement of a simulated contaminated wound, with the aim of possible future application in clinical settings.

## Methods

### Study design and participants

Our study protocol was approved by our local ethics committee (Ethics committee University Medical Center Freiburg, Freiburg, Germany). All human participants provided written informed consent. No trials on live animals were performed; therefore no permission for animal trials was required. All experiments were conducted according to the ethical standards determined by the Declaration of Helsinki 1964 and its later additions."

All human trials were performed by medical professionals experienced in wound debridement who provided written informed consent to participate. In robotic trials, wound registration to the robotic system was performed by one experienced surgeon in accordance with the instructions of an engineer who developed the robotic system.

### Surgical robotic system

**Fig 1:**
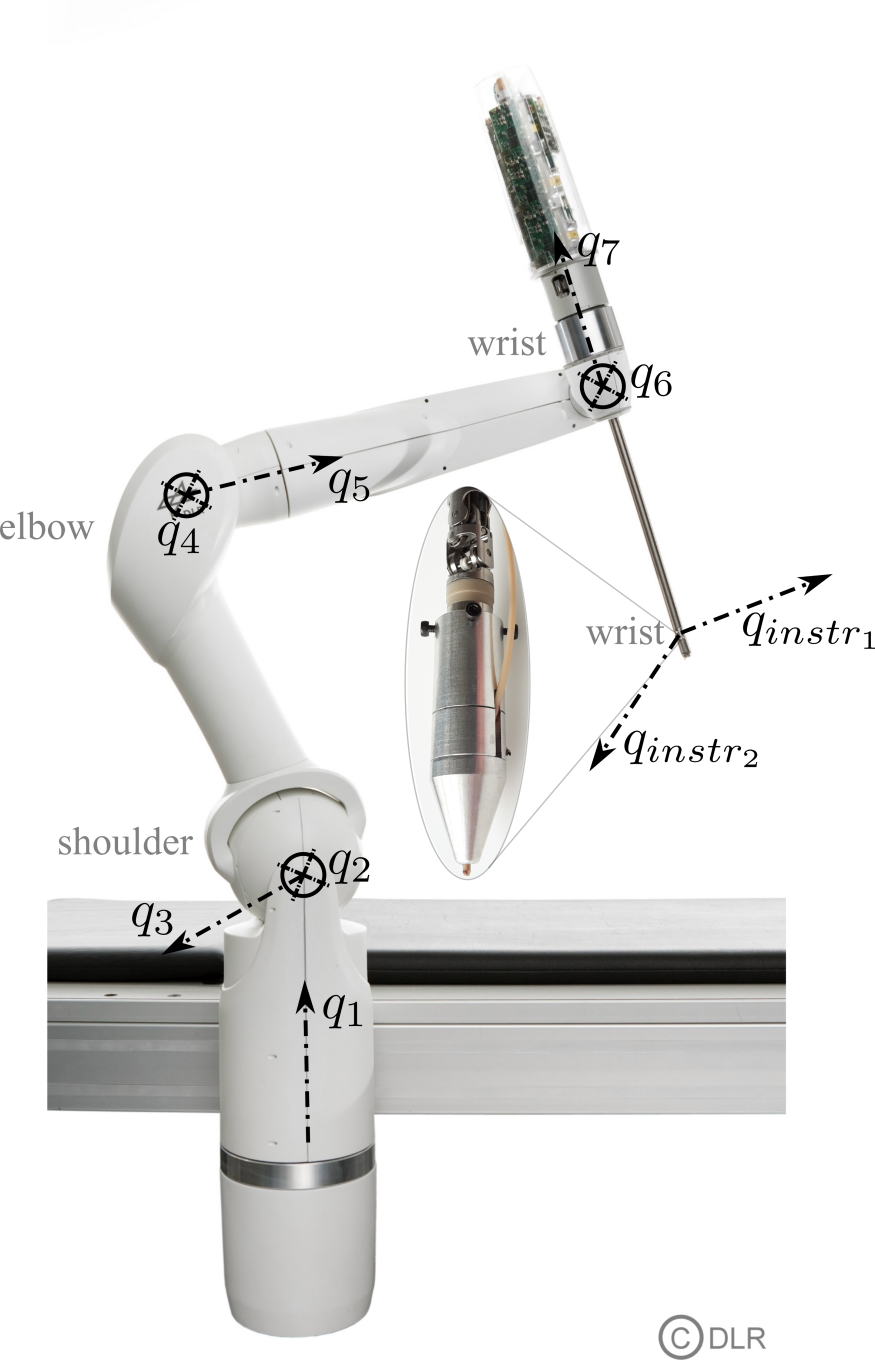
DLR MIRO and DLR MICA with axis.

The robotic system used was based on the DLR MIRO, a 7 degree of freedom (DoF) versatile lightweight robot (Fig 1 *q*_1_–*q*_7_) [8]. Unlike dedicated robotic systems developed for a single application, the DLR MIRO is designed to meet the requirements of various surgical applications, as demonstrated by several benchmark applications including pedicle screw setting in orthopedic spine surgery [10], biopsy collection [11], internal mammary artery detection [12], and as the MiroSurge platform for minimally invasive surgery [13]. The equivalence of the DLR MIRO on the instrument side is the DLR MICA, a versatile instrument for robotic surgery consisting of a drive unit that can be equipped with task-specific tools [14]. It provides a 2 DoF wrist (Fig 1 $q_{{instr}_{1}},q_{{instr}_{2}}$) and an additional functional DoF, which was not utilized here. The DLR MICA is mounted to the hollow shaft wrist of the DLR MIRO (Fig 1). In combination, this setup provides 9 DoF, enabling the system to reach any pose (position and orientation) within a dedicated task space (e.g. minimally invasive or traditional tasks) in its entire workspace. Six DoF enable full manipulability inside or outside the human body. The remaining three DoF can be utilized to adapt to spatial limitations within the crowded operation room environment without affecting the motion of the tool or to implement semi-autonomous subtasks.

### Adaption of robotic system for waterjet actuation

**Fig 2:**
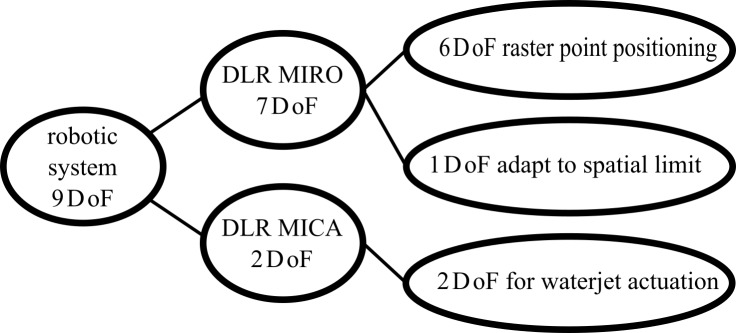
Utilization of the 9 degrees of freedom in the waterjet wound debridement task.

The robotic system consisting of the DLR MIRO and DLR MICA meets the requirements to handle a waterjet resulting from its physical behavior, guaranteeing the best cutting effect, as described before [9]. The utilization of the available DoF for the waterjet wound debridement task is depicted in Fig 2. To mount the flexible waterjet probe to the DLR MICA, an adapter was developed (see center region of Fig 1). Thus, the flexible waterjet probe can be actuated by the 2 DoF ($q_{{instr}_{1}},q_{{instr}_{2}}$) provided by the DLR MICA to clean the wound surface. Additionally, the waterjet system was integrated into the real-time communication infrastructure of the robotic system to control activation of the waterjet as well as the suction device via software.

### Waterjet wound debridement

All robotic and human control trials were performed using an ERBEJET 2 device (ERBE Elektromedizin GmbH, Tuebingen, Germany), which provides a high powered punctual waterjet beam parallel to the handle and orthogonal to the tissue. The pressure of the waterjet is generated by a sterile single-use double-piston pump. pressure range with a 120-μm jet nozzle is 1–80 bar (100–8,000 kPa) with a volume flow of 1–55 ml/min as a laminar liquid jet. The ERBEJET 2 provides a versatile waterjet surgery platform for various applications in open as well as in endoscopic surgery [15, 16].Water pressure was set to 50 bar. Suction for all trials was performed with a separate, hand-guided suction device. For the robotic trials, a flexible endoscopic waterjet probe was utilized as described above, for all human trials a hand applicator was used. The geometry of the nozzle of the hand applicator as well as the flexible probe is identical, providing a comparable waterjet beam. The function of the waterjet was not altered for this study and is identical to the clinically used device.

### Porcine skin wound model

To evaluate the feasibility of a robotically supported waterjet system for surgical wound debridement, an *ex vivo* porcine skin model containing subcutaneous tissue as well as muscle was employed for all trials. All animal material originated from domestic livestock and was provided by a local meat processing facility. Porcine skin samples were acquired and used at the day of slaughter. The three-dimensional shape of the simulated wound was determined by the anatomy of the porcine skin sample, and samples were classified according to their surface curvature into 4 categories: flat, convex, concave, and inclined. Two different wound shapes were simulated by cutting a standardized form into the porcine skin with a wound depth of 0.8 cm. Wound shapes utilized in the experiments were a round wound with a diameter of 6 cm, a square with 8 cm edges, and a concave side with circle radius of 2 cm. Depth and size of all wounds were controlled after cutting using a master template to ensure a high degree of standardization. The time required for wound cleaning was recorded for all trials. Completeness of coverage and off-target areas were recorded as percentage of total wound area. Detection of areas covered by the waterjet scalpel was performed by using colored oil paint (MUSSINI, H. Schmincke & Co. GmbH & Co. KG, Erkrath, Germany) in robotic trials and invisible UV light color (neon nights UV-Farben, Electronic Commerce Global Ltd., Hong Kong) in human trials to avoid confounding. The waterjet scalpel leaves traces in the paint covered areas and can therefore be detected. The choice of color was determined and validated by a pretrial prior to this study, using a variety of different color indicators as well as other substances. The size of the areas not covered by the waterjet and off-target areas were then determined using photo-documentation of all wound areas and the open-source image processing software ImageJ to calculate the relative size of areas with no waterjet pattern within the color coding.

### Statistical evaluation

Statistical analysis was performed using the Statistical Package for Social Sciences software (SPSS version 20.0, IBM Corp., Armonk, NY). Differences between groups were analyzed by Kruskal-Wallis test for non-parametric data and, in case of significant differences, confirmed by the Mann-Whitney test. For numeric data, differences were analyzed by ANOVA and, in case of significance, confirmed by t test. P values < 0.05 were considered significant. All data are presented as mean ± standard deviation.

## Results

### Evaluation of human approach to wound debridement using simulated wounds on paper

To evaluate the human approach to wound cleaning regarding pattern and approach prior to the waterjet trials five human subjects were given the task to completely cover a square-shaped area with one ellipsoid side without affecting boundaries. The task was to simulate waterjet debridement with a UV light pen by handling the pen like a waterjet scalpel with oscillating movements. The square had either a side length of 8 cm or 5 cm; for both sizes, 5 trial runs were performed by each participant. Drawing pattern and time were recorded for each trial, and off-target area and missed area were analyzed using photodocumentation as described. Average time was 136.56 ± 77.89 s for the smaller area and 178.8 ± 69.3 s for the larger area. The average area not covered by UV light color was 1.58% ± 1.3% for the smaller simulated wound area, and significantly larger (12.21% ± 9.38%; p = 0.000) for the larger area. The variation across trials was substantial, ranging from full coverage to 4.45% missed area in the case of the small square and to 30.36% in the large square. The average area the participants covered outside the defined wound area was 0.27% ± 0.85% (range, 0 to 3.27%) for the smaller wound and 0.23% ± 0.75% (range, 0 to 3.66%) for the larger area. There was no significant difference in off-target area between the large and the small areas (p = 0.395). The participants demonstrated significant differences in performance with regard to time (p = 0.001), uncovered area (p = 0.016), and off-target area (p = 0.00). All participants used a meandering pattern starting in one corner of the area. One participant used a circular pattern, starting in the center of the wound, but refrained from using this approach again after one trial. There was no significant difference in missed coverage or off-target area for this approach.

### Evaluation of human performance in waterjet wound debridement using a porcine skin wound model

To have a benchmark for our robotic trials, 5 different human participants operating a waterjet scalpel as described above, were given the task to perform wound debridement on a porcine skin wound model on two different simulated wounds. Drawing pattern and procedure time were recorded for each trial, and off-target area and missed area were analyzed using photodocumentation as described above. In the first series of trials, all participants cleaned a standardized round wound shape with 5-cm diameter. Average time spent on each wound was 98.52 ± 21.73 s, and average missed area was 4.85% ± 4.09% (range, 0.77% to 18.15%). The average area cleaned outside of the defined area was 1.84% ± 1.25% (range, 0.21% to 5.44%). A second series of trials included a standardized square wound shape with 8-cm side length and one ellipsoid side. Average cleaning time for this wound was 133.50 ± 45.27 s (range, 63 s to 300 s). The average missed area was 6.92% ± 4.36% (range, 0.9% to 17.33%), and the area cleaned outside the outlines of the wound was 1.79% ± 1.18% (range, 0.45% to 5.67%). All trials were repeated 10 times. The porcine skin wound models included 3 different type of surface curvature: 10 inclined wounds, 10 convex wounds, 10 concave wounds, and 20 flat wounds for each wound size, each of them equally distributed between the participants. No significant differences in time (p = 0.134 [small wound]; p = 0.726 [large wound]), missed area (p = 0.244 [small wound]; p = 0.267 [large wound]), or off-target area (p = 0.296 [small wound]; p = 0.811 [large wound]) were observed across the different wound shapes. However, the participants demonstrated high variability in performance, with the average covered wound area ranging from 90.9% for the worst performer to 95.8% for the highest-performing participant. Individual participants also demonstrated high variability across trials, with area not covered ranging from 1.2% to 17.1% in the participant with the highest fluctuation. On observation of the cleaning pattern, all participants started in one corner, utilizing meandering patterns but refraining from a detectable systematic cleaning pattern shortly after the start of the trial.

### Workflow development for semiautonomous performance of wound debridement using robotic waterjet actuation

To perform wound debridement using robotic waterjet actuation a workflow had to be developed for the robotic system. To overcome human limitations regarding variability and accuracy, a semiautonomous approach was chosen and implemented into the robotic control system. The workflow follows the sense–plan–act (SPA) paradigm, a common approach used in robotics [17]. It consists of the following three steps:

1. Sense: Set up a world model based on measured values.
2. Plan: Plan a specific task based on the registered world model.
3. Act: Execute the planned task.

The SPA workflow is controlled by the surgeon by providing the sampled data for the first step or evaluating the performed execution to either continue or repeat the task at hand. The complete SPA process as well as the interaction with the surgeon is implemented within a graphical user interface combined with a virtual reality environment, so that the registered world model as well as each performed step can be observed and controlled by the user. The steps developed for the waterjet wound debridement were defined as follows.

Sense: Since the DLR MIRO provides adequate accuracy, it is used in hands-on mode (gravity compensated) as an input device for haptic sampling to generate a point-cloud representing the wound area. Here the surgeon touches the area to be treated with the hand-guided robot and records the samples with a foot pedal. This method reduces the complexity of the system and its set-up, since the robot is used as an input and output device. No further sensory input (e.g. camera or tracking device) is necessary. The result is a point cloud as starting point for the planning step.

Plan: In this step, the point-cloud that specifies the target area is used to first build a surface model (S1 Text) of the wound and then perform the path planning based on the generated model. To approximate the target area, a paraboloid surface function is used, since it considers curvature in two directions. This approach guarantees good approximations of flat areas as well as complex geometries, as found for example at the knee, elbow, shoulder blade or at the transition from the extremities to the trunk. The surface model is achieved through the process of approximation, comprising multiple steps. A more detailed description of this step including the underlying mathematical model can be found under S1 Text. First, the point-cloud is locally approximated by a plane, which serves as the base coordinate system for further calculation of a paraboloid target function. With this surface model, arbitrary points lying on the wound area can be calculated which further enables a rasterization of the area. The result is a convex polygon representing the wound area (Fig 3.1) within a rectangular shaped rasterized area extending over the borders of the wound. The rectangle is subsequently rasterized numerically in equidistant spaces in the x- and y-directions (Fig 3.2). Then, the raster points are labeled as inside or outside of the convex polygon. The polygon is further approximated by patches. Depending on the patch size and the overlapping strategy chosen by the surgeon, a subset of the raster points is selected (Fig 3.3). Based on this model the generation of a driving path for the robot for the cleaning process is now possible and a meander path in the x-y direction is built, considering only the subset of raster points that demonstrate the “inside the convex polygon” attribute (Fig 3.4). Fig 4 shows an example convex polygon with its meander path and the applied raster projected onto the x-y plane. The target area is approximated with surface patches at each subset raster point on the meander path. The patch shape is chosen as a Lissajous curve according to Bahls et al. [9], since this approach offers a high grade of coverage in adequate treatment time with half-patch overlapping. The gray shading depicts the density of the coverage in a certain region. The inner region shows the highest coverage. The edge regions show less coverage, since it is assumed that the target area was defined with ample margins, and the outline of the polygon is located in the healthy area. In most cases, this approach is acceptable, since the selective effect of the waterjet leaves the healthy tissue unharmed [18]. Nevertheless, in some cases the introduction of “prohibited areas” may be necessary, e.g. in cases of artificial stoma or open adnominal wounds with incomplete fascial closure [19, 20]. In such cases, the waterjet is limited to the inner area, and crossing of the convex polygon outline is not allowed. The presented workflow also considers this issue. Therefore, the convex polygon is shrunk by shifting each parallel edge using half of the square’s diagonal (Fig 5). Hence, the inner region can be treated according to the approach presented above. Additionally, the edge area is treated separately by using an adapted patch shape along a path between the outer and inner polygon (corner points marked by yellow dots in Fig 5).

**Fig 3:**
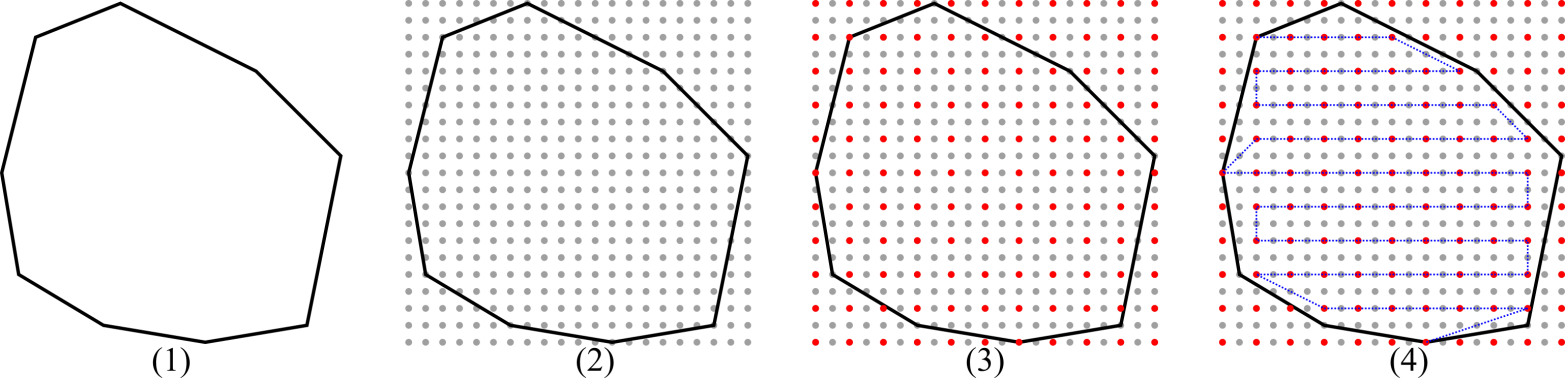
Steps of the path planning of the target area modeled as a convex polygon projected on an x-y plane.

**Fig 4:**
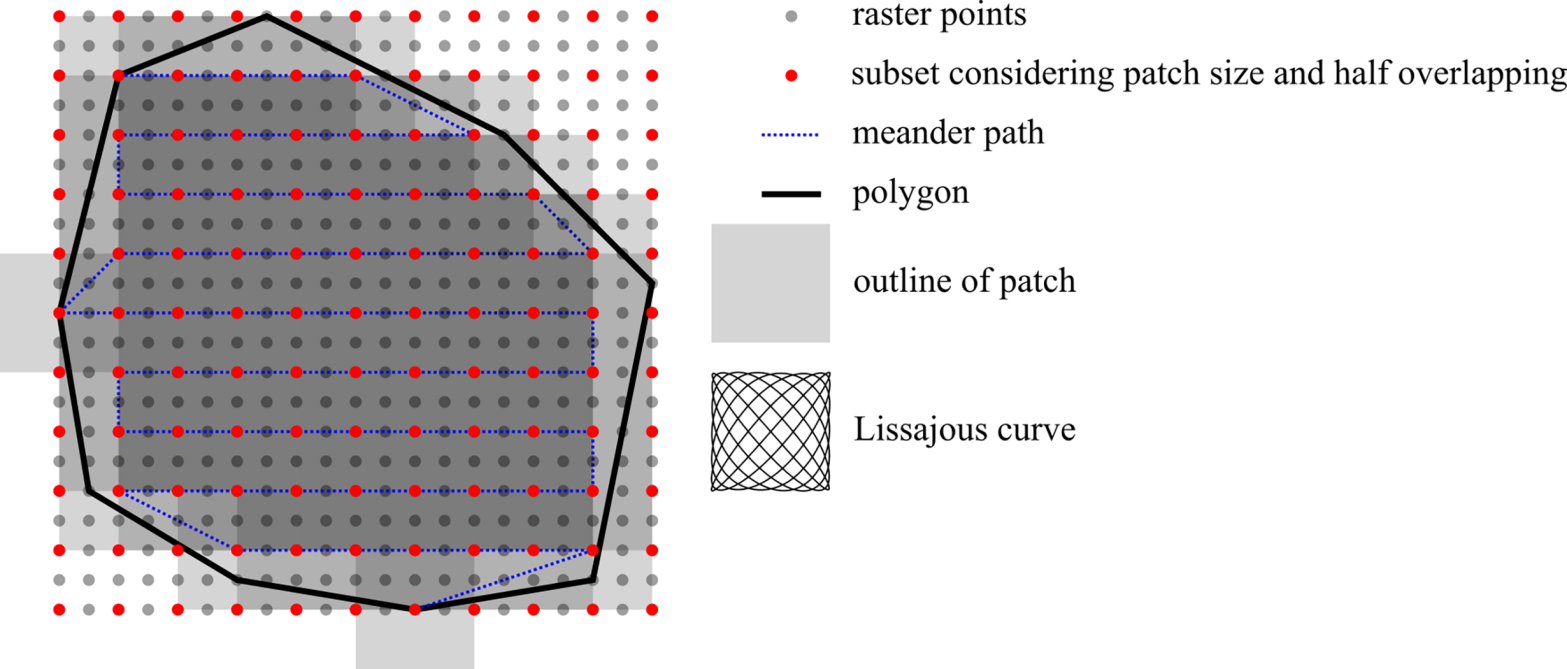
Convex polygon projected on an x-y plane with rasterized rectangular area, grade of coverage, and planned path.

**Fig 5:**
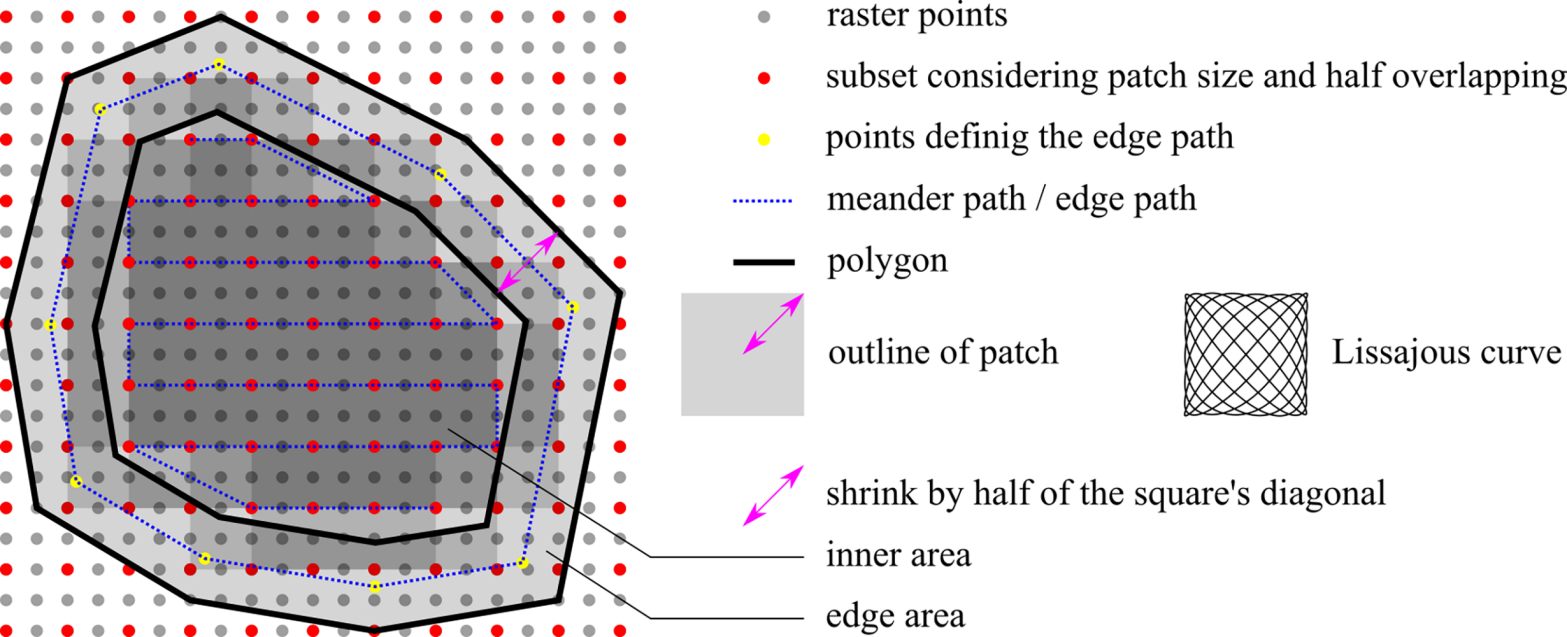
Convex polygon under consideration of “prohibited areas”.

Act: The starting point of this step is an ordered list of poses representing a meander path covering the target area. If crossing of the sampled area is not allowed, two lists are used representing the inner and edge area. First, the surgeon guides the robot in “hands-on mode” to a valid starting pose, which guarantees sufficient space between the instrument and the tissue to be treated. The surgeon then starts the process, and the robot automatically moves the instrument equipped with the waterjet probe to the first pose. At this point, the specified Lissajous curve (covering the patch size, Fig 5) is followed by the nozzle of the waterjet. The activation and deactivation of the waterjet and the suction are respectively triggered. The system successively moves the instrument to the next pose and treats the related patches with the waterjet. After performing the full meander path, the surgeon decides to either stop the process or repeat the act step with the same set of poses. In the event that certain areas require additional treatment, resampling can be performed to generate a new set of poses. The optional handling of the edge areas, which guarantees not crossing the outline of the convex polygon, is performed in the same manner.

## Evaluation of robotic performance in waterjet wound debridement using a porcine skin wound model

Trials using the robotic system were performed on wounds of two different shapes and sizes. All trials, including the registration of the wound area and activation of the robotic system, were performed by a medical professional without previous experience using the system. The first trial utilized a round wound shape with wounds having a 5-cm diameter. The cleaning was repeated on 10 different wounds with different surface curvatures, and average procedure time was 470.3 ± 19 s (range, 435 s to 493 s). The area not cleaned by the robotic system was 3.14% ± 2.55% (range, 0% to 7.88%). The average cleaned area exceeding the wound boundary was 1.33% ± 1.41% (range, 0% to 3.95%). All wounds were categorized according to their surface curvature, with 3 flat, 3 inclined, 2 convex, and 2 concave wounds included. No significant differences in time (p = 0.405), missed area (p = 0.707), or off-target area (p = 0.481) were observed across the different wound shapes. The number of registered points in the wound area and the wound edge were recorded. On average, 25.6 points were registered for the outer edges, and 24.6 points were registered for the inner surface of the wound. The number of registered points was not correlated with outcome. For a second set of trials, a standardized square wound shape with 8-cm side length and one ellipsoid side was used, and trials were repeated 10 times. The wounds were categorized according to their surface curvature, including 2 concave, 3 inclined, 3 flat, and 2 convex wounds. For this wound shape, no significant differences in time (p = 0.611), missed area (p = 0.423), or off-target area (p = 0,744) were observed across the different wound shapes. The average time for cleaning was 885.33 ± 102.07 s (range, 723 s to 1033 s). The average area not cleaned by the robotic system was 3.57% ± 2.16% (range, 0% to 5.98%). The average cleaned area exceeding the wound boundary was 2.81% ± 2.56% (range, 0% to 9.46%).

## Comparison of human and robotic performance in waterjet wound debridement using a porcine skin wound model

The average missed area over all trials was 6.23% ± 6.1% (range, 0% to 30.36%) for the human control group and 2.86% ± 2.38% (range, 0% to 7.88%) for robotically cleaned wounds, indicating a significant advantage for the robotic trials (p = 0.031). By contrast, the average cleaned area exceeding the wound boundaries was 1.29% ± 1.32% (range, 0% to 5.67%) for human trials and 2.07% ± 2.24% (range, 0% to 9.46%) for robotic trials, indicating a non-significant trend toward advantage in the human group (p = 0.087). The human group demonstrated a highly significant advantage in procedure time (p = 0.001).

## Discussion

Waterjet wound debridement is a widely used method for wound debridment [21]. Wound bed preparation has been a common medical problem and multiple novel approaches have been proposed and shown promising results. A recent study suggested electrical plasma dissection for surgical treatment of chronic ulcers [22] showing a high efficiency. Additionally, the vacuum wound sealing technique has been shown to positively affect wound healing, leading to a faster reduction in wound area compared to conventional methods [23]. Furthermore, ultrasound waves have been suggested to show a beneficial effect on wound healing when delivered to the wound area through a fluid mist [24]. While all these modern techniques have been shown to be superior to conventional methods [25], a recently performed systematic review detected a benefit of waterjet wound debridement especially in infected[26] and fibrinous wounds[27]. In this study,we evaluated a novel system for wound debridement using a robotic waterjet platform for automated actuation of the waterjet scalpel. Often, devices with a large contact area of water scalpel and skin, for example the Versajet hydrosurgery system (Smith & Nephew, London, UK), are used, but punctual waterbeam systems are also available, for example debritom+ (Medaxis AG, Baar, Switzerland) and provide higher precision due to their smaller field of impact. This is especially important in situations, where vital tissue is adjacent to the wound area and needs to be preserved. Examples include parastomal wounds [28] or situations with open abdominal wounds and exposed intestine [29] as well as situations in which skin graft has been performed and remaining exposed wound areas need to be cleaned or a skin graft is intended and wound area needs to be kept as small as possible. In our study, a punctual waterjet scalpel was utilized, focusing on precision as well as wound coverage; both parameters were evaluated. While not specifically designed for wound debridement, the ERBEJET 2 device provides a high powered punctual waterjet beam capable of sufficiently cutting tissue. For our study, this device was chosen due to its high adaptability, provided by a variety of available applicators.

The clinical value of robotic waterjet wound debridement was then assessed by simulating various wound characteristics that frequently occur in clinical practice. Currently, a variety of experimental models are available, including silicon-based models and hydrogels [30], that show similar behavior to human skin tissue. However, all models have significant limitations with regard to wound simulation, since they were designed to simulate the intact dermal surface. Since the behavior of various tissues under waterjet treatment has been previously investigated, our main focus was to evaluate the potential of a combination of waterjet and robotic system to automatically perform adequate wound debridement. We chose an *ex vivo* porcine skin model to simulate wounds of different sizes, shapes, and surface curvatures. To assess the quality of wound debridement, we used oil paint for robotic trials and UV-light paint for human trials. The suitability of this approach was verified by comparing different methods of recording areas covered by the waterjet scalpel when cleaning a surgical wound. While camera-based approaches were excluded, due to view obstruction by the water, various wound coatings were examined. Oil paint as well as UV light paint allowed a reliable detection of cleaned areas as well as indicating areas affected by the waterjet outside the limit established by the wound edges in preliminary trials. When evaluating the robotic system, our data demonstrated that the selected parameters and overlapping strategy of the Lissajous curves cover more than 96% of the target area. The low standard deviation suggests that this approach offers good reproducibility independent of wound shape and surface curvature. The average wound coverage achieved by humans was significantly lower than that of the robot, and the standard deviation of human trials was almost twice that achieved by the robot. The apparent advantages of a robotic system, i.e. repeatability and consistent accuracy independent of any effect due to fatigue [31], are obvious. The robot is capable of performing the procedure with very little variation in results, potentially leading to a lower number of necessary treatments and achieving a higher quality of wound conditioning with faster healing. The cleaning of off-target areas was roughly comparable between human and robotic trials. However, while the violation areas outside the wound borders by human participants were sporadically distributed over the whole course of the wound, the mistakes made by the robotic system were systematic and mostly caused by concave wound edges, which cannot be included in the current mathematical model, and changes in wound form during the procedure, caused by a slight edematous swelling of the tissue triggered by the water treatment. Further improvements, such as incorporating concave areas in the path generation and adjusting the defined path during the cleaning procedure in adaptation to wound shape changes, will optimize the current system, providing a further clinical advantage over human performance. These additions are also necessary to increase the capabilities of the current technological state of the robotic system. Complicated wounds, such as wound pockets, deep wounds with high edges, and wounds around sensitive areas (e.g. parastomal wounds) were purposefully excluded from the current study, since they overstrain the functions of the present system. To overcome these challenges, more user interaction in the path generation process is necessary to give the system the required information to include problematic areas such as wound pockets as well as exclude sensitive areas when calculating the path of the robotic arm during debridement. The implementation of these additional features is already in progress, and through the inclusion of specific modalities, such as a “wound edge mode,” the system will be able to meet these clinical challenges. While these limitations preventing the present system from being ready for application in clinical settings, the usability of the current prototype is already very high.

All robotic trials were performed by a trained medical professional after only a short introduction to the system by the developing engineers. The use of the system was therefore intuitive, and path generation and debridement execution were initiated without any necessity of technical support by the developing engineers. However, a clear disadvantage of the robotic system is the significantly increased procedural time. While the recorded period only included the actual time of performing debridement, the disadvantage is even greater when considering the additional time necessary to outline the wound shape to the robotic system. The evaluation of time however, is of only minor importance for various reasons. First, the required treatment time should be considered over the whole course of clinical treatment necessary to achieve sufficient wound conditioning. Since the completeness of coverage is directly related to the necessity for repeated treatments [32], a higher quality of performance by the robotic system might decrease the overall time of treatment by decreasing the number of necessary interventions, although single procedures take longer. This improved performance may also lead to an economic benefit, as treatment of chronic wounds often incurs significant cost [33]. In addition, the current system, designed for maximum coverage, uses a predetermined number of treatment cycles without considering the state of the wound and performs debridement without real-time evaluation of the result. By contrast, human trials were always stopped when the participant considered the wound sufficiently cleaned. Possible improvements to the robotic system include visual inspection of cleaning progress by the surgeon, who can stop the treatment based on their experience. Autonomous camera-based inspection could also be considered. Finally, the shorter treatment time of human participants is in large part due to the DLR MICA robotic system used in this trial. Its main design goal was telerobotic manipulation, which requires high manipulation force instead of high actuation frequency. With adapted hardware, higher actuation frequencies are possible, which could substantially reduce the treatment time of the robotic procedure.

Our current data shows that the implementation of robotic assistance in waterjet wound debridement can improve both the efficiency and quality of surgical wound conditioning, potentially providing a genuine advantage for patients with chronic infected wounds. In addition, the concept provides benefits for surgeons and medical professionals, since implementation of the robotic system allows semiautonomous performance of the waterjet application, leading to a reduction in necessary personnel as well as significantly reduced risk of cross-infection However, our study also revealed significant limitations that must be considered. The current wound model cannot replace experiments on *in vivo* tissue, since dead tissue may not respond to water treatment in the same way as live tissue. Wound deformation due to edema as well as partial uptake of color used to track the cleaning pattern are also potential cofounding factors in the present study. In addition, suction for robotic trials was performed manually, since an actuated waterjet probe with integrated suction is not yet commercially available, although such a device is under development. This issue could also create variation in the measured results. With integrated suction, residual water and consequent damping of the jet can be further reduced, which may be expected to further improve results.

## Conclusion

Robotic waterjet wound debridement is a promising new technological approach compared to the current clinical standard of surgical wound conditioning. Despite some limitations, our data indicate greater quality and efficiency of the robotic system compared to humans. With high ease of use as well as a significant advantage for infection safety among operating personnel, the current prototype shows high potential for clinical application. However, further refinements, such as the implementation of more user feedback to allow the debridement of complicated wound shapes and greater procedural adaptability, are still necessary. After implementation of these improvements, trials on more complicated wounds as well as *in vivo* models are necessary to more thoroughly evaluate the clinical potential of this technology before its appliance in routine patient care.

## Supporting information

S1 Text Mathematical approach to derive the surface model. (DOCX)
