# Supplementary material for: Robotic waterjet wound debridement – Workflow adaption for clinical application and systematic evaluation of a novel technology
Source: PLoS One. 2018 Sep 27;13(9):e0204315. doi: 10.1371/journal.pone.0204315 (PMC6160027; doi:10.1371/journal.pone.0204315)
Supplement: S1 Text — (DOCX) [file pone.0204315.s001.docx]

**S1 Text: Mathematical approach to derive the surface model**

The derivation of the surface model is achieved by first locally approximating the point-cloud by a plane [[1](#_ENREF_1)], which serves as the base coordinate system for the second-order analytic function

|  | $f\left( xy \right)=z=ax^{2}+bxy+cy^{2}+dx+ey+f .$ | (1) |
| --- | --- | --- |

After transforming all recorded samples into this new coordinate system, the paraboloid target function (1) can be approximated. Therefore, equation (1) is applied to all recorded and transformed samples of the point-cloud. This results in a set of linear equations described in matrix representation with

|  | $Ar=s .$ | (2) |
| --- | --- | --- |

Where

|  | $A=\left( \begin{matrix} {x_{0}}^{2} & x_{0}y_{0} & {y_{0}}^{2} & x_{0} & y_{0} & 1 \\ {x_{1}}^{2} & x_{1}y_{1} & {y_{1}}^{2} & x_{1} & y_{1} & 1 \\ \vdots& \vdots& \vdots& \vdots& \vdots& \vdots\\ {x_{n}}^{2} & x_{n}y_{n} & {y_{n}}^{2} & x_{n} & y_{n} & 1 \end{matrix} \right) ,$ | (3) |
| --- | --- | --- |

the coefficients of the target function are described with

|  | $r=\left( \begin{matrix} a \\ b \\ c \\ d \\ e \\ f \end{matrix} \right)$ | (4) |
| --- | --- | --- |

and

|  | $s=\left( \begin{matrix} z_{0} \\ z_{1} \\ \vdots\\ z_{n} \end{matrix} \right) .$ | (5) |
| --- | --- | --- |

Since the number of equations exceeds the number of unknowns represented by the vector $r$, equation (2) is overdetermined, and therefore the system cannot be solved directly by standard methods, e.g. Gaussian elimination. Hence, to obtain a solution for the system of equations, a least-squares approach is applied. The least squares method is “*a mathematical procedure for finding the best-fitting curve to a given set of points by minimizing the sum of the squares of the offsets ("the residuals") of the points from the curve. The sum of the squares of the offsets is used instead of the offset absolute values because this allows the residuals to be treated as a continuous differentiable quantity*” [[1](#_ENREF_1)]. Applying this procedure to (2) leads to

|  | $A^{T} A\hat{r}=A^{T}s$ | (6) |
| --- | --- | --- |

whereas $\hat{r}$ is the best approximated $r$ [[2](#_ENREF_2)], such that all coefficients of (1) are determined.

**References:**

1. Weisstein EW. Least Squares Fitting. From MathWorld 2016. Available from: http://mathworld.wolfram.com/LeastSquaresFitting.html.

2. G S. Introduction to linear algebra. : Wellesley-Cambridge Press; 2016.
